# Sequential Modulations in a Combined Horizontal and Vertical Simon Task: Is There ERP Evidence for Feature Integration Effects?

**DOI:** 10.3389/fpsyg.2017.01094

**Published:** 2017-06-30

**Authors:** Katharina Hoppe, Kristina Küper, Edmund Wascher

**Affiliations:** Leibniz Research Centre for Working Environment and Human Factors (IfADo), TU Dortmund, DortmundGermany

**Keywords:** Simon task, sequential modulation, action control, conflict adaptation, feature integration, ERP

## Abstract

In the Simon task, participants respond faster when the task-irrelevant stimulus position and the response position are corresponding, for example on the same side, compared to when they have a non-corresponding relation. Interestingly, this Simon effect is reduced after non-corresponding trials. Such sequential effects can be explained in terms of a more focused processing of the relevant stimulus dimension due to increased cognitive control, which transfers from the previous non-corresponding trial (conflict adaptation effects). Alternatively, sequential modulations of the Simon effect can also be due to the degree of trial-to-trial repetitions and alternations of task features, which is confounded with the correspondence sequence (feature integration effects). In the present study, we used a spatially two-dimensional Simon task with vertical response keys to examine the contribution of adaptive cognitive control and feature integration processes to the sequential modulation of the Simon effect. The two-dimensional Simon task creates correspondences in the vertical as well as in the horizontal dimension. A trial-by-trial alternation of the spatial dimension, for example from a vertical to a horizontal stimulus presentation, generates a subset containing no complete repetitions of task features, but only complete alternations and partial repetitions, which are equally distributed over all correspondence sequences. In line with the assumed feature integration effects, we found sequential modulations of the Simon effect only when the spatial dimension repeated. At least for the horizontal dimension, this pattern was confirmed by the parietal P3b, an event-related potential that is assumed to reflect stimulus–response link processes. Contrary to conflict adaptation effects, cognitive control, measured by the fronto-central N2 component of the EEG, was not sequentially modulated. Overall, our data provide behavioral as well as electrophysiological evidence for feature integration effects contributing to sequential modulations of the Simon effect.

## Introduction

Stimulus–response compatibility (SRC) paradigms like the Simon task (Simon, 1969) are helpful research tools to investigate action control. In the Simon task, participants are asked to respond to non-spatial stimulus features (e.g., color or letter-identity), which are mapped onto spatially arranged response keys. Although the spatial position of the stimuli is explicitly irrelevant for the task, participants usually respond faster and more accurately when response location and task-irrelevant stimulus location are spatially corresponding compared to trials in which they are not (Simon and Rudell, 1967; Simon, 1969; Lu and Proctor, 1995). The term “Simon effect” is primarily used with respect to response times (RT). Effects of the spatial S–R correspondence are also evident in other variables like accuracy or ERPs. For the sake of clarity, we will further call these effects Simon-like or (spatial) correspondence effects. Simon (1969, p. 174) explained the Simon effect with a “natural tendency to respond toward the source of stimulation”. The irrelevant stimulus dimension activates a corresponding response (Kornblum et al., 1990), which can be confirmed by the lateralized readiness potential (LRP; see Coles, 1989) of the electroencephalogram (EEG) (e.g., De Jong et al., 1994; Wascher et al., 2001; Stürmer et al., 2002). Dual-route models (Kornblum et al., 1990; De Jong et al., 1994) assume that S–R transmission in the Simon task proceeds via a direct and an indirect route. While the direct route operates via automatic processing of the irrelevant spatial stimulus information, the indirect route involves controlled processing of the task-relevant stimulus features. It follows that both routes activate the same response in corresponding trials, whereas they activate opposing response tendencies in non-corresponding trials. The prolonged RT observed in non-corresponding Simon trials are assumed to result from this response conflict (Kornblum et al., 1990; De Jong et al., 1994).

Interestingly, trial-by-trial analyses have shown that the Simon effect is more pronounced after corresponding than after non-corresponding trials indicating that the magnitude of the Simon effect is also modulated by the S–R correspondence of the preceding trial (e.g., Stürmer et al., 2002; Hommel et al., 2004; Wühr, 2005; Wühr and Ansorge, 2005). These sequential modulations are often attributed to an adaptation of cognitive control (Botvinick et al., 2001; Stürmer et al., 2002). Such conflict adaptation account posits that the response conflict associated with non-corresponding Simon trials requires an increased recruitment of cognitive control processes, which can carry over to the subsequent trial and thus modulate the magnitude of the Simon effect. To be more precise, Stürmer et al. (2002) proposed that cognitive control mechanisms are utilized to close the direct S–R transmission path in non-corresponding trials resulting in a diminished influence of task-irrelevant spatial stimulus information. This suppression of task-irrelevant information leads to a reduced response conflict and shortened RTs in subsequent non-corresponding trials (n–N sequences, whereby the first, small letter indexes the previous trial correspondence and the second, capital letter the current trial correspondence of a trial sequence). RTs will instead be increased in subsequent corresponding trials (n–C sequences) as the suppressed spatial information no longer reinforces the response tendency activated by the direct S–R transmission path. As a result, the Simon effect is reduced after non-corresponding compared to after corresponding trials (c–C and c–N sequences) (Stürmer et al., 2002).

An alternative explanation of the sequential modulation of the Simon effect is the feature integration account (Hommel, 1998; Hommel et al., 2004; for a similar account see Mayr et al., 2003). It is assumed that in experimental trials, stimulus and response features are bound into event files (Hommel, 1998). Consecutive trials can thus form three different types of sequences: complete repetitions, partial repetitions and complete alternations of S–R bindings. In complete repetition sequences of the Simon task, two consecutive trials are identical as to stimulus identity/response position (both features are confounded) and stimulus position. In the current trial, the event file of the preceding trial is therefore re-activated, thus facilitating performance. In partial repetition sequences, only some but not all trial features are repeated. The event file created on the preceding trial thus does not match the requirements of the current trial. It is nevertheless re-activated by the task feature(s) shared by the two trials necessitating an unbinding process, which impedes performance. Finally, in complete alternation sequences, all task features change, but performance is not distorted, as no time-consuming unbinding process is needed (Hommel, 1998; Hommel et al., 2004). In a two-choice Simon task, complete alternations and complete repetitions occur only if the correspondence condition does not change (i.e., in c–C and n–N sequences), whereas a change of the correspondence condition (i.e., in n–C and c–N sequences) always leads to a partial repetition of task features.

Hence, both the conflict adaptation account and the feature integration account can explain the improved performance in c–C and n–N compared to c–N and n–C sequences. The former sees an adaptation of cognitive control mechanisms as causal, the latter the degree of task-feature repetitions and many studies tried to disentangle their contribution to sequential modulations in SRC paradigms (e.g., Hommel et al., 2004; Wühr, 2005; Wühr and Ansorge, 2005; Akçay and Hazeltine, 2007; Chen and Melara, 2009; Spapé et al., 2011). In line with the conflict adaptation account, Wühr (2005), for example, reported sequential modulation effects with different correspondence sequences even when the amount of feature changes was kept constant. On the other hand, there is also evidence supporting feature integration effects. Hommel et al. (2004, Experiment 3) found sequential modulations of the Simon effect even when subjects were instructed not to respond in the first of two consecutive trials, which contradicts the assumption of a response conflict as the causal factor. Previous research thus has provided empirical evidence in favor of both conflict adaptation and feature integration effects contributing to sequential modulations of the Simon effect. Both accounts (feature integration and conflict adaptation) are not necessarily mutually exclusive. Abstract learning mechanisms of for example conflict-related processes and concrete learning mechanisms of task features for example might interact to determine the magnitude of sequential effects (Verguts and Notebaert, 2008; Weissman et al., 2015). Furthermore, trial-by-trial RT carry-over effects might also play a role in modulating correspondence effects sequentially (cf. Huber-Huber and Ansorge, 2016). As it remains unclear to which extent different mechanisms contribute to behavior in a given situation we focus on investigating conflict adaptation and feature integration processes in one paradigm. One possible approach to tease apart the distinct contributions of these two mechanisms is to examine ERP components of the EEG in the Simon task, which several earlier studies with similar research questions did not (e.g., Wühr, 2005; Wühr and Ansorge, 2005; Akçay and Hazeltine, 2007). In contrast to behavioral measures, ERP measures allow us to examine the impact of sequential modulations on distinct processing stages due to the high temporal resolution of the EEG.

At an electrophysiological level, the fronto-central N2 has been used to investigate sequential modulation effects in SRC paradigms (e.g., Wendt et al., 2007; Chen and Melara, 2009; Clayson and Larson, 2011). This ERP component has a negative peak at fronto-central electrodes around 250 ms after stimulus onset and has been identified as a correlate of cognitive control processes (e.g., Folstein and Van Petten, 2008) and the detection of response conflicts (Wendt et al., 2007). The conflict adaptation account assumes a lower conflict after non-corresponding trials. The preceding conflict boosts cognitive control and the closing of the direct path increases the focus on the target, that is decreases the influence of the irrelevant information. On the other hand, after corresponding trials, the direct path stays open since its information is beneficial on corresponding trials. Hence, the focus on the target is low and the flow of irrelevant information is not reduced in the following trial (Botvinick et al., 2001; Stürmer et al., 2002). Research has shown that currently non-corresponding trials lead to increased fronto-central N2 amplitudes in the Simon task (Chen and Melara, 2009) and other SRC tasks (Wendt et al., 2007; Clayson and Larson, 2011). Importantly, while excluding S–R repetition trials, that is while controlling for repetition priming effects (Mayr et al., 2003), fronto-central N2 amplitudes were smallest for n–C sequences and successively increased for c–C, n–N, and c–N sequences (Clayson and Larson, 2011) providing support for the conflict adaptation account.

Given that they may index cognitive control processes, frontro-central N2 modulations may, however, not be very well suited to assess feature integration effects (but see Chen and Melara, 2009). A more appropriate measure may be modulations of the parietal P3b amplitude, which, to our knowledge, have not been examined in the context of sequential modulations in the Simon task, as of yet. The P3b is a posterior ERP component with a positive peak around 400 to 450 ms (in visual tasks). It is assumed to be evoked by memory retrieval mechanisms related to the evaluation of stimuli, which require some kind of action (Donchin et al., 1986; for a review see Kok, 2001). It has been argued that these memory processes may be related to response set selection (Hillyard and Kutas, 1983). More recently, Verleger et al. (2014) claimed that P3b amplitude reflects the reactivation of S–R links in terms of Hommel’s (1998) event files. These claims have to be taken with caution, however, since they cannot fully explain expectancy effects on P3b amplitude (Verleger and śmigasiewicz, 2016), and the effects of other factors, such as task difficulty, which may overlie effects of S–R linking on P3b amplitude (Kok, 2001). Research concerning the modulation of the P3b amplitude on current trials in the Simon task is also sparse and results are rather mixed (e.g., Ragot and Renault, 1981; Valle-Inclán, 1996). Whereas Ragot and Renault (1981) did not find a spatial correspondence effect on the P3b amplitude, Valle-Inclán (1996) observed a larger P3b amplitude on trials with corresponding S–R relations compared to trials with non-corresponding S–R relations.

**FIGURE 1:**
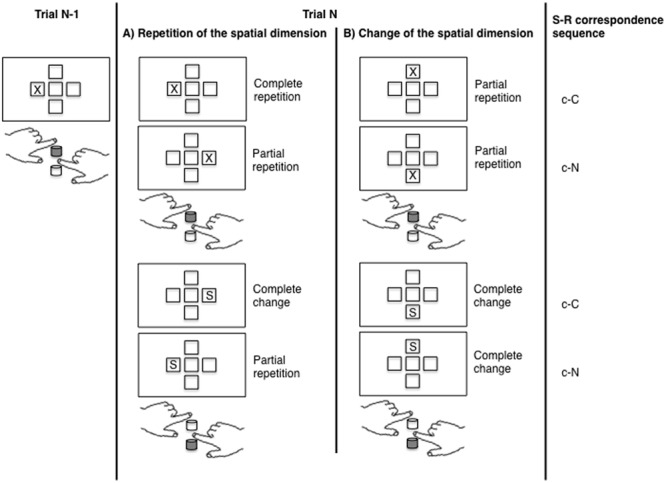
The figure shows different trial sequences of the Simon task with a dimensional repetition **(A)** and a dimensional change **(B)**. Depicted are different trial types (trial N) following a horizontal corresponding trial (trial N-1). For each trial sequence, degree of event file overlap (complete repetition, partial repetition, complete change) and S–R correspondence sequence (c = corresponding, n = non-corresponding, small letters indicating previous trial characteristics, capital letters current trial characteristics) are indicated. In this example, the upper response key was assigned to the X stimulus and the left hand, whereas the lower key was assigned to the S stimulus and the right hand. The correct response key for each trial is colored in gray.

In the present study, we investigated the contribution of conflict adaptation and feature integration effects on sequential modulations of the Simon effect by using different spatial dimensions (cf. for example Wühr, 2005). In the standard one-dimensional Simon task, trial sequences in terms of Hommel (1998; Hommel et al., 2004) account, that is complete repetitions, complete alternations and partial repetitions of task features, are confounded with sequences of S–R correspondence (see **Figure 1A**). This means that unbinding processes are only needed in sequences where the S–R correspondence changes (c–N, n–C). When the S–R correspondence repeats (c–C, n–N) on the other hand there are no partial repetitions und thus no unbinding of event files is necessary. Due to this confound it is challenging to dissociate between feature integration and conflict adaptation effects as both accounts lead to the same predictions regarding the behavioral outcomes of the different conditions. By adding a second spatial dimension, for example vertical stimulus positions, to the horizontal dimension, this problem can be solved (**Figure 1B**, cf. for example Wühr, 2005). Importantly, whenever the spatial dimension changes from one trial to the next, unbinding processes are possible in every S–R correspondence sequence (c–C, n–N, c–N, n–C). This design thus creates novel trial types which resolve the confound between the necessity for unbinding processes and S–R correspondence changes inherent in the one-dimensional two-choice Simon task. Previous studies which used two spatial dimensions in the Simon task either implemented a task-switching paradigm (Braem et al., 2011) or used the second spatial dimension as a means of creating specific types of stimuli that allowed to force unbinding processes when the correspondence condition repeated (e.g., Wühr, 2005). Both approaches potentially add some processing necessities to the task, which are not directly related to the question of sequential effects in the Simon task itself.

Thus, in the present study, we presented a Simon task in both the vertical and the horizontal dimension. Due to vertically positioned response keys the S–R correspondence was also implemented with respect to both spatial axes. During the given trial, one of two possible target stimuli appeared either on the horizontal axis (left or right from the center of the screen) or on the vertical axis (above or below the center of the screen). Irrespective of the position of the stimulus, one stimulus (e.g., the letter X) was mapped to the upper response key (e.g., pressed with the left hand) and the other stimulus (e.g., the letter S) was mapped to the lower response key (e.g., pressed with the right hand). To increase the similarity between spatial dimensions and avoid the implementation of task-switching elements (cf. Egner, 2008; Braem et al., 2014), S–R mapping was the same for the vertical and the horizontal dimension. It is important to note that this approach might promote joint representations of S–R links across spatial dimensions that might otherwise (i.e., when stimuli on different dimensions are associated with different responses) not exist. We deemed equivalent S–R mapping across dimensions necessary for the current task, however, as distinct S–R mappings for each dimension would have drawn undue attention to the spatial position of stimuli, which, per definition, should be irrelevant in the Simon task.

Since a change of the spatial dimension, that is from a vertical to a horizontal stimulus presentation (v–H sequence, whereby the first, small letter indexes the previous spatial dimension and the second, capital letter the current spatial dimension in a trial sequence) or vice versa (i.e., h–V sequence), always leads to an alternation of the stimulus position, the study design generates a subset containing no complete repetitions of task features, but only complete alternations and partial repetitions, which are equally distributed over all correspondence sequences. Thus, whenever the spatial dimension changes from one trial to the next, unbinding processes due to partial repetitions can come to bear in any kind of correspondence sequence (c–C, c–N, n–C, n–N) with the same probability (**Figure 1B**). As displayed in **Figure 1**, a trial sequence, in which an X is displayed on the left side is followed by an X displayed above the screen center, reflects a partial repetition as both trials contain the exact same stimulus as well as the same response, whereas the position of the stimulus (which is irrelevant for the task) is changed. Although the sequence reflects a partial repetition, the correspondence condition may repeat. On the other hand, when the spatial dimension repeats, the situation resembles a standard two-dimensional Simon task. In this second subset of trials, partial repetitions and in turn unbinding processes are only evident, when the correspondence condition changes (i.e., in c–N and n–C sequences). Complete changes or complete repetitions, which do not require unbinding processes, are only evident, when the correspondence condition repeats (**Figure 1A**). Note that in both subsets, that is in sequences in which the spatial dimension changes and in sequences in which it repeats, the absolute amount of partial repetitions is the same, but is no longer confounded with the correspondence sequence. Although there is some debate as to whether the horizontal Simon and the vertical Simon effect rely on the same mechanisms (Wiegand and Wascher, 2005; Vallesi et al., 2005), previous studies provide evidence for a similar, albeit smaller Simon effect in the vertical dimension as in the horizontal dimension (Nicoletti and Umiltà, 1984). In keeping with this, Valle-Inclán (1996) found a similar result pattern on the P3b in a vertical and a horizontal Simon task. The vertical Simon effect has also been used to study conflict adaptation effects (e.g., Stürmer et al., 2002) and it seems reasonable to similarly use it to investigate the impact of feature integration and conflict adaptation mechanisms on sequential effects (cf. Wühr, 2005).

In the present study, we examined whether sequential modulations of the Simon effect (RT) and of spatial correspondence effects on accuracy and ERPs occur irrespective of a trial-by-trial change of the spatial dimension or whether such effects are only evident in trials with an unbalanced proportion of unbinding processes (i.e., only when the spatial dimension repeats). Results from previous research concerning similar questions were rather mixed as sequential effects across spatial dimensions were found in some studies (Braem et al., 2011), but not in others (Lee and Cho, 2013). We thus aimed to further investigate sequential modulation effects in an SRC task using ERP measures in order to identify possible underlying mechanisms. For the current experimental design, the conflict adaptation and the feature integration account arrive at distinct predictions regarding the result pattern.

According to the conflict adaptation account (Botvinick et al., 2001; Stürmer et al., 2002), the size of the Simon effect should be modulated by the preceding correspondence condition – the Simon effect should thus be larger after corresponding compared to after non-corresponding trials. On the premise that the conflict adaptation effect affects behavior in a general way, a change of the spatial dimension should not affect these sequential modulations. In other words, when a horizontal non-corresponding trial “closes” the direct route, this should affect subsequent horizontal as well as vertical trials and vice versa. On the other hand, the feature integration account (Hommel et al., 2004) predicts that there should be no sequential modulations of the Simon effect in sequences when the spatial dimension changes, as the probability for partial repetitions is the same for any S–R correspondence sequence (c–C, c–N, n–C, n–N). However, as long as stimuli vary only within one dimension, as is the case in the standard spatially one-dimensional Simon task, the well-known sequential effects should be observed and should be comparable across spatial dimensions. To assess cognitive control (Folstein and Van Petten, 2008) and S–R link processes (e.g., Verleger et al., 2014), which might reflect feature integration effects, we analyzed the fronto-central N2 and P3b components of the EEG, respectively. For the fronto-central N2 component with its assumed sensitivity to conflict adaptation processes, we expected that it is modulated by correspondence sequence when these effects are due to conflict adaptation (cf. Clayson and Larson, 2011). These conflict adaptation effects should be evident irrespective of a repetition or alternation of the spatial dimension. To assess the contribution of feature integration effects, we further evaluated the parietal P3b. Unbinding processes likely require the reset of the S–R mapping and hence should affect the efficiency of response selection processes (cf. Verleger et al., 2014). Feature integration effects might therefore sequentially modulate the P3b amplitude. In this case, the result pattern should be similar to the pattern posits by the feature integration account with respect to the behavioral data (see above).

To sum up, within each spatial dimension, we expected to observe similar sequential modulations as in the standard one-dimensional Simon task. Spatial correspondence effects should be smaller, eliminated or reversed after non-corresponding trials, as predicted by both the conflict adaption and the feature integration account. When a change of the spatial dimensions occurs in the trial sequence, both accounts predict different outcomes. According to the feature integration account, sequential effects should be eliminated, that is the size of the Simon effect should be independent from the S–R correspondence of the previous trial. The conflict adaptation account on the other hand predicts the same pattern of results regardless the spatial relation of two subsequent trials.

## Materials and Methods

### Participants

Twenty-nine right-handed participants (14 female) took part in the experiment for payment or course credit. Because of technical problems (4) and wrong instructions (1) 5 participants (2 female) were excluded from further analysis. The age of the remaining 12 female and 12 male participants ranged from 18 to 30 years (*M* = 24). All subjects provided informed written consent according to the Declaration of Helsinki, had normal or corrected-to-normal vision and were naïve as to the aim of the experiment. No participant reported neurological or psychiatric diseases. The ethics committee of the Leibniz Research Centre for Working Environment and Human Factors approved the study.

### Stimuli and Apparatus

Participants were seated in an armchair in a sound-attenuated, electrically shielded, and dimly lit chamber. The stimuli were displayed on a 100 Hz CRT monitor (22-inch, 20 inch/51 cm viewable) with a resolution of 1024 × 768. The viewing distance was 120 cm. Task programming was conducted with Lazarus IDE (Lazarus Team, 1993–2016) and the presentation of the visual stimuli was controlled by a VSG 2/5 graphic accelerator (Cambridge Research Systems, Rochester, UK). Stimuli and display background were both colored in shades of gray (color spectrum CIE 1932, *x* = 0.287, *y* = 0.312) with different luminance values (stimuli: 45 cd/m^2^, background: 10 cd/m^2^). The stimuli thus appeared to be lighter than the background.

**FIGURE 2:**
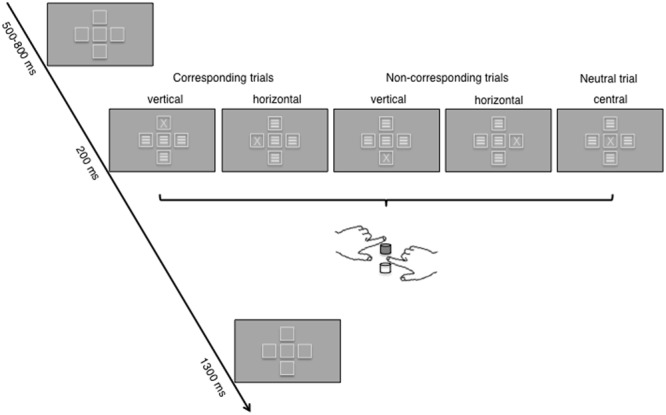
The figure shows the time course of a trial in the two-dimensional Simon task; the example used depicts trials with an X target. For each of the five possible stimulus positions, S–R correspondence (corresponding, non-corresponding, neutral) according to hand positioning and S–R mapping for one of the four counterbalanced groups is indicated (upper key: left hand, X; lower key: right hand, S). The correct response key under these instructions is colored in gray. Frames at the five positions were visible throughout the trial. During target presentation, X or S appeared in one frame while the other frames were filled with noise stimuli (three lateral bars).

The letters X and S were chosen as target stimuli in the Simon task to guarantee that targets were as symmetrical as possible and had little to no symbolic or literal association to the vertical or horizontal dimension. Five frames, each with a visual angle of 1° (frame lines = 0.05°), were displayed during the whole experiment and functioned as placeholder for the appearance of the stimuli. The frames formed a plus symbol, with four frames surrounding the frame in the middle of the display (**Figure 2**). The vertical and lateral distance from the central screen point to the four surrounding frames had a visual angle of 1.5° each. This arrangement resulted in two different spatial dimensions where the target stimuli could appear – horizontal (to the left or right of the central frame) and vertical (below or above the central frame). Furthermore, as part of a control condition, the target stimuli could appear within the central frame which had no obvious relation to the two spatial dimensions. The presentation of the two target stimuli at the five stimulus positions was randomized and equally distributed across trials. On each given trial, either an X or an S appeared in one of the five frames. In all remaining frames, noise stimuli were presented in order to avoid asymmetrical perceptual input, which can distort the recorded EEG data (see for example Wascher and Wauschkuhn, 1996). Noise stimuli consisted of three lateral bars (visual angle was 0.08° each) in the center of the frames.

The response panel consisted of two vertically arranged force keys (vertical distance: 10 cm), which were positioned exactly above each other (**Figure 2**). To minimize visual information about the participant’s hand placement, the response panel was positioned as close as possible to the participant’s torso. The button presses were executed by the index fingers of the left and the right hand. Participants’ exerted force values were recorded along with the raw EEG signal. Response parameters (force values, RTs, accuracy) were calculated online from the VSG input to monitor participants’ performance.

Thus, in each trial, except for those in the neutral condition, correspondence occurred either with respect to the hand (left/right) or with respect to the response key (up/down). On horizontal corresponding trials, stimulus position and response hand were on the same side (e.g., left hand and left stimulus position), whereas on horizontal non-corresponding trials, stimulus and response hand were on opposite sides (e.g., left hand and right displayed stimulus). On vertical corresponding trials, stimulus and response key were either both up or down (e.g., upper button and stimulus above the central frame), whereas on vertical non-corresponding trials stimulus and response key were in opposite directions (e.g., upper button and stimulus under the central frame or vice versa). Importantly, irrespective of the spatial dimension of the stimulus the instruction was kept the same within and across all experimental blocks. Consequently, there was no task switching and the response set was kept unchanged.

### Procedure

The experimental phase consisted of three blocks with 600 trials each, which each took about 27 min to complete. After each block, there was a 5 min break before the next block was initiated. Hence, there were 1800 trials in total per participant. Consequently, each trial condition (combination of S–R correspondence and spatial dimension: vertical corresponding (vc), vertical non-corresponding (vn), horizontal corresponding (hc), horizontal non-corresponding (hn), and central was presented 360 times during the experiment. Each sequence condition (hc–HC, hc–HN, hc–VC, hc–VN, hn–HC, etc., whereby the first, small letters index the previous trial type and the second, capital letters index the current trial type of a trial sequence) was presented 72 times.

Prior to the experimental phase, participants received written instructions and performed 100 practice trials (approximately 5 min. duration), which were not included in the data analysis. Participants were asked to fixate the frame in the middle of the screen throughout each experimental block. The instructions emphasized both speed and accuracy. During the experimental phase, participants did not receive any feedback about their performance quality. Half of the participants were told to press the upper key with the index finger in response to the letter X and the lower key in response to the letter S, whereas the other half of participants received the opposite S–R mapping rule. Within each of these S–R mapping rule groups, half of the participants had to place their left hand on the upper plane of the response panel and the right hand on the lower plane, and vice versa. Thus, there was a completely counterbalanced experimental setting, but each participant had a fixed S–R mapping throughout the whole experiment. Participants were instructed to ignore the position of the target and respond to the mapping of the stimulus type (X or S) and the hand position (upper or lower key). The instructions thus emphasized the vertical dimension, but did not allude to the horizontal dimension.

One trial lasted between 2000 to 2300 ms in total (**Figure 2**). During the whole experimental phase, the five placeholder frames were visible. Hence, the first display in each trial consisted of these frames and was presented for a variable time between 500 and 800 ms. Subsequently, one target and four noise stimuli were displayed for 200 ms in the centre of the five frames. After 1300 ms the next trial was initiated.

### Data Analysis

Electroencephalogram data processing and analyses as well as the analyses of the response force were conducted with MATLAB^®^ 2013b (Mathworks Inc., Natick, MA, United States). For the EEG data, we additionally used the MATLAB^®^ toolboxes EEGlab 13.4.3B (Delorme and Makeig, 2004) and ERPlab 4.0.3.1 (Lopez-Calderon and Luck, 2014). Statistical analyses were performed with R 3.1.2 in RStudio (RStudio, Inc.). In a first step, data were collapsed across blocks and experimental settings.

### Behavioral Data

For every participant, an individual force threshold value was calculated as the maximal force exerted across all given responses. If the force of a button press exceeded 5% of this individual maximal force value it was counted as a response. RT was thus defined as the time when 5% of the individual maximal force value was exceeded. If the force value exceeded 30% of the individual maximal force within the next 200 ms, the button press was counted as a complete response, otherwise it was counted as a partial response. Correct responses were defined as complete responses within a time range of 150 to 1500 ms after stimulus onset and which were in accordance with the instructions. As we did a sequential analysis of the data, we only included trials after correct responses in our data analyses. Error trials included trials with misses, false responses with respect to the S–R mapping, responses outside the time range and partial responses, which were not followed by a complete response.

### EEG Recording

The EEG was recorded from 60 Ag/AgCI active scalp electrodes (ActiCap; Brain Products, Gilching, Germany), which were mounted in an elastic cap according to the extended 10/20 System (Pivik et al., 1993). During the EEG recording, a BrainAmp DC amplifier (Brain Products, Gilching, Germany) with 250 Hz low-pass filtering and a sampling rate of 1000 Hz was used. The ground electrode was affixed at FPz. The online reference electrode was placed at P9. To measure and control for eye movements, an electrooculogram (EOG) was recorded. Two electrodes were placed above and below the right eye (vertical EOG) and two electrodes were fixed at the outer canthi of each eye (horizontal EOG). Impedances, that is the resistance between skin and electrode, were controlled and kept at less than 10 kΩ.

### EEG Preprocessing

EEG data were re-referenced offline against the mastoids (TP9, TP10). Afterward, broken channels were rejected. EEG data were then filtered with a band pass filter ranged from 1 to 15 Hz. As we analyzed the EEG data in a stimulus-locked fashion, the data were then segmented into intervals of 2200 ms (–700 to 1500 ms after stimulus onset) and the baseline was set to a 200 ms interval (–200 to 0 ms) prior to the stimulus presentation. For artifact correction, an independent component analysis (ICA) was performed and the ADJUST function (Mognon et al., 2011) was used to detect artifacted ICs. After this, the IC structure was written into the 1000 Hz data and artefactual ICs were removed. The data were then segmented again into intervals of 2100 ms (–700 to 1400 ms after stimulus onset) and the baseline was set at a 200 ms interval (–200 to 0 ms) prior to stimulus presentation. Epochs with artifacts were excluded. Finally, rejected channels were interpolated.

### EEG Measurements

In the present study, we analyzed the fronto-central N2 and the parietal P3b ERP components. For the evaluation of the fronto-central N2 component, the peak EEG amplitude was measured in the time interval of 200–330 ms after stimulus onset at FCz. Due to high inter-individual variability of the maximum peak, we used the mean amplitude between 390 and 450 ms after stimulus presentation at Pz instead of the peak amplitude as a measurement for the parietal P3b.

### Statistics

For the dependent behavioral variables, that is RT and accuracy, as well as for the ERP components fronto-central N2 and P3b, separate repeated measures analysis of variance (ANOVA) were conducted including the within-subjects factors S–R correspondence on current trials (*corr N*: corresponding versus non-corresponding), spatial dimension on current trials (*spat N*: vertical versus horizontal), S–R correspondence on previous trials *(corr N-1*: corresponding versus non-corresponding) and spatial dimension on previous trials (*spat N-1*: vertical versus horizontal). For *post hoc* comparisons, reduced ANOVAs were conducted, which only included the factors of interests. These reduced ANOVAs were only performed when appropriate interactions were found in the higher-order ANOVAs. The analyses of the EEG data as well as of the RT data were conducted only for trials, which featured a correct response and were additionally preceded by correct trials. The alpha level was set at 5% and partial η^2^ are mentioned as a measure of effect size.

For RTs, we additionally conducted a separate analysis of the neutral condition (i.e., target presentation at the central stimulus position), which functioned as a control condition. To this end, a subset was created, which only included the neutral stimulus position in current trials. Two one-factorial ANOVAs were conducted with the previous trial type as the within-subject factor. As only sequences in which a neutral trial is followed by another neutral trial had no assumed unbinding process, the first ANOVA included the neutral condition on trial N-1 as a factor level, whereas in the second ANOVA this factor level was excluded. No further analyses of the neutral condition were conducted.

## Results

### Behavioral Data

#### Response Times

**FIGURE 3:**
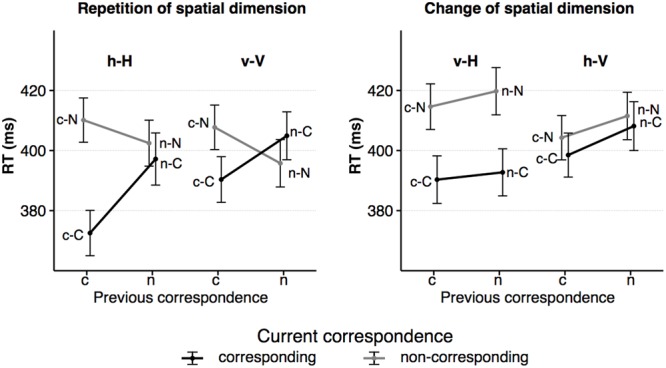
The figure shows the average RTs as a function of dimensional repetition **(left)** and dimensional change **(right)** as well as current and previous S–R correspondence (sequence types: c–C, n–C, c–N, n–N). Error bars indicate the standard error of the mean. h = horizontal dimension, v = vertical dimension, c = corresponding S–R relation, n = non-corresponding S–R relation. Small letters indicate previous trial features, capital letters current trial features of a trial sequence.

The RT data are illustrated in **Figure 3**. The overall ANOVA revealed a significant main effect of the factor *corr N* with faster responses in corresponding compared to non-corresponding trials, *F*(1,23) = 54.43, *p* < 0.001, $\eta_{p}^{2}$ = 0.70. This pattern is consistent with the Simon effect. Other main effects were found for *spat N, F*(1,23) = 5.50, *p* = 0.028, $\eta_{p}^{2}$ = 0.19, and for *corr N-1, F*(1,23) = 37.19, *p* < 0.001, $\eta_{p}^{2}$ = 0.62. RTs were shorter on current horizontal trials compared to on current vertical trials and preceding corresponding trials resulted in faster responses than preceding non-corresponding trials. No main effect was found for *spat N-1, F*(1,23) = 1.62, *p* = 0.215, $\eta_{p}^{2}$ = 0.07.

The interaction of *corr N* and *corr N-1, F*(1,23) = 50.15, *p* < 0.001, $\eta_{p}^{2}$ = 0.69, indicated that the size of the Simon effect was sequentially modulated by the previous correspondence condition. After corresponding trials, the Simon effect was more pronounced, *F*(1,23) = 117.88, *p* < .0001, $\eta_{p}^{2}$ = 0.84, compared to after non-corresponding trials, *F*(1,23) = 8.07, *p* = 8.07, $\eta_{p}^{2}$ = 0.26. The *corr N* by *spat N* interaction indicated that the size of the Simon effect was also affected by the spatial dimension, *F*(1,23) = 32.40, *p* < 0.001, $\eta_{p}^{2}$ = .59, in that it was more pronounced in the horizontal dimension, *F*(1,23) = 61.50, *p* < 0.001, $\eta_{p}^{2}$ = 0.73, compared to the vertical dimension, *F*(1,23) = 4.95, *p* = 0.036, $\eta_{p}^{2}$ = 0.18. Furthermore, the *spat N* by *spat N-1* interaction showed an increase in RT after a change of the spatial dimension, *F*(1,23) = 53.25, *p* < 0.001, $\eta_{p}^{2}$ = 0.70. This increase in RT was larger after horizontal, *F*(1,23) = 48.80, *p* < 0.001, $\eta_{p}^{2}$ = 0.68, compared to after vertical trials, *F*(1, 23) = 8.41, *p* = 0.008, $\eta_{p}^{2}$ = 0.27. The fourfold interaction of the factors *corr N, spat N, corr N-1* and *spat N-1, F*(1,23) = 38.14, *p* < 0.001, $\eta_{p}^{2}$ = 0.62, showed that sequential modulations of the Simon effect were only evident in sequences without a change of the spatial dimension (h–H sequences: *F*(1,23) = 39.32, *p* < 0.001, $\eta_{p}^{2}$ = 0.63; v–V sequences: *F*(1,23) = 35.86, *p* < 0.001, $\eta_{p}^{2}$ = 0.61), but not when the spatial dimension changed (v–H sequences: *F*(1,23) < 1; h–V sequences: *F*(1,23) < 1). In h–H sequences, the Simon effect was evident only after corresponding, *F*(1,23) = 104.32, *p* < 0.001, $\eta_{p}^{2}$ = 0.82, but not after non-corresponding trials, *F*(1,23) < 1. In v–V sequences, the Simon effect was found after corresponding trials, *F*(1,23) = 26.77, *p* < 0.001, $\eta_{p}^{2}$ = 0.54, and a reversed Simon effect was found after non-corresponding trials, *F*(1,23) = 6.33, *p* = 0.019, $\eta_{p}^{2}$ = 0.22 (see **Figure 3**).

Neither of the other interactions reached statistical significance (*spat N* × *corr N-1*: *F*(1,23) < 1, *corr N* × *spat N-1*: *F*(1,23) < 1, *corr N-1* × *spat N-1*: *F*(1,23) = 3.99, *p* = 0.058, $\eta_{p}^{2}$ = 0.15, *corr N* × *spat N* × *corr N-1*: *F*(1,23) < 1, *corr N* × *spat N* × *spat N-1*: *F*(1,23) = 1.13, *p* = 0.298, $\eta_{p}^{2}$ = 0.05, *corr N* × *corr N-1* × *spat N-1*: *F*(1,23) = 1.88, *p* = 0.184, $\eta_{p}^{2}$ = 0.08, *spat N* × *corr N-1* × *spat N-1*: *F*(1,23) < 1).

### Response Accuracy

The main effect of *corr N* indicated a spatial correspondence effect on accuracy, *F*(1,23) = 22.60, *p* < 0.001, $\eta_{p}^{2}$ = 0.50; participants responded more accurately in corresponding trials (*M* = 0.94, *SE* = 0.00) compared to non-corresponding trials (*M* = 0.91, *SE* = 0.01). The interaction of *corr N* and *corr N-1* furthermore signals a sequential modulation of the spatial correspondence effect on task accuracy, *F*(1,23) = 35.60, *p* < 0.001, $\eta_{p}^{2}$ = 0.61. Similarly to the RTs, the spatial correspondence effect on accuracy was only evident after corresponding, *F*(1,23) = 32.56, *p* < 0.001, $\eta_{p}^{2}$ = 0.59 (c–C: *M* = 0.95, *SE* = 0.01 versus c–N: *M* = 0.90, *SE* = 0.01), but not after non-corresponding trials, *F*(1,23) = 3.64, *p* = 0.069, $\eta_{p}^{2}$ = 0.14 (n–C: *M* = 0.94, *SE* = 0.01 versus n–N: *M* = 0.92, *SE* = 0.01). Again, as in the RT data, the fourfold interaction of *corr N, corr N-1, spat N* and *spat N-1, F*(1,23) = 23.00, *p* < 0.001, $\eta_{p}^{2}$ = 0.50, indicated that this sequential modulation of the spatial correspondence effect on accuracy was only evident when there was no trial-to-trial alternation of the spatial dimension (h–H sequences: *F*(1,23) = 47.17, *p* < 0.001, $\eta_{p}^{2}$ = 0.67; v–V sequences: *F*(1,23) = 19.71, *p* < 0.001, $\eta_{p}^{2}$ = 0.46), but it was not significant when the spatial dimension changed (v–H sequences: *F*(1,23) < 1; h–V sequences: *F*(1,23) < 1). Within each spatial dimension, the well-known sequential modulation was found: In h–H sequences, the spatial correspondence effect on accuracy only emerged after corresponding trials, *F*(1,23) = 36.76, *p* < 0.001, $\eta_{p}^{2}$ = 0.62 (c–C: *M* = 0.97, *SE* = 0.00 versus c–N: *M* = 0.88, *SE* = 0.02), but not after non-corresponding trials, *F*(1,23) = 1.07, *p =* 0.313, $\eta_{p}^{2}$ = 0.04 (n–C: *M* = 0.93, *SE* = 0.01 versus n–N: *M* = 0.94, *SE* = 0.01). In v–V sequences, a spatial correspondence effect was also only evident after corresponding trials, *F*(1,23) = 11.78, *p =* 0.002, $\eta_{p}^{2}$ = 0.34 (c–C: *M* = 0.96, *SE* = 0.01 versus c–N: *M* = 0.90, *SE* = 0.02), but not after non-corresponding trials, *F*(1,23) < 1 (n–C: *M* = 0.93, *SE* = 0.01 versus n–N: *M* = 0.93, *SE* = 0.01). For accuracy, neither further main effects (*spat N*: *F*(1,23) < 1, *corr N-1*: *F*(1,23) = 1.13, *p* = 0.299, $\eta_{p}^{2}$ = 0.05, *spat N-1*: *F*(1,23) = 3.62, *p* = 0.07, $\eta_{p}^{2}$ = 0.14) nor interactions (*corr N* × *spat N*: *F*(1,23) < 1, *spat N* × *corr N-1*: *F*(1,23) < 1, *corr N* × *spat N-1*: *F*(1,23) = 1.34, *p* = 0.259, $\eta_{p}^{2}$ = 0.06, *spat N* × *spat N-1*: *F*(1,23) = 1.31, *p* = 0.263, $\eta_{p}^{2}$ = 0.05, *corr N* × *spat N*: *F*(1,23) = 1.01, *p* = 0.326, $\eta_{p}^{2}$ = 0.04, *corr N* × *spat N* × *corr N-1*: *F*(1,23) < 1, *corr N* × *spat N* × *spat N-1*: *F*(1,23) < 1, *corr N* × *corr N-1* × *spat N-1*: *F*(1,23) = 1.88, *p* = 0.182, $\eta_{p}^{2}$ = 0.08, *spat N* × *corr N-1* × *spat N-1*: *F*(1,23) < 1) reached statistical significance.

### RT Analysis in Neutral Trials

The one-factorial ANOVA revealed that on current central trials the preceding trial type had a statistically significant influence on the RT, *F*(4,92) = 15.64, *p* < 0.001, $\eta_{p}^{2}$ = 0.40, with the averaged shortest RTs in current neutral trials following neutral trials (*M* = 365.15 ms, *SE* = 7.78 ms) and longer RTs in current neutral trials following other trial types. Current neutral trials lead to the following RTs after horizontal corresponding trials: *M* = 379.23 ms, *SE* = 7.55 ms, after horizontal non-corresponding trials: *M* = 383.76 ms, *SE* = 7.99 ms, after vertical corresponding trials: *M* = 378.19 ms, *SE* = 7.85 ms and after vertical non-corresponding trials: *M* = 379.79 ms, *SE* = 7.81 ms. In a second step, we conducted a separate one-factorial ANOVA and removed the neutral condition on trial N-1. In line with the feature integration account, after this removal the sequential modulation disappeared, *F*(3,69) = 1.92, *p* = 0.132, $\eta_{p}^{2}$ = 0.08.

### Electrophysiological Data

#### Fronto-Central N2 Peak Amplitude

**FIGURE 4:**
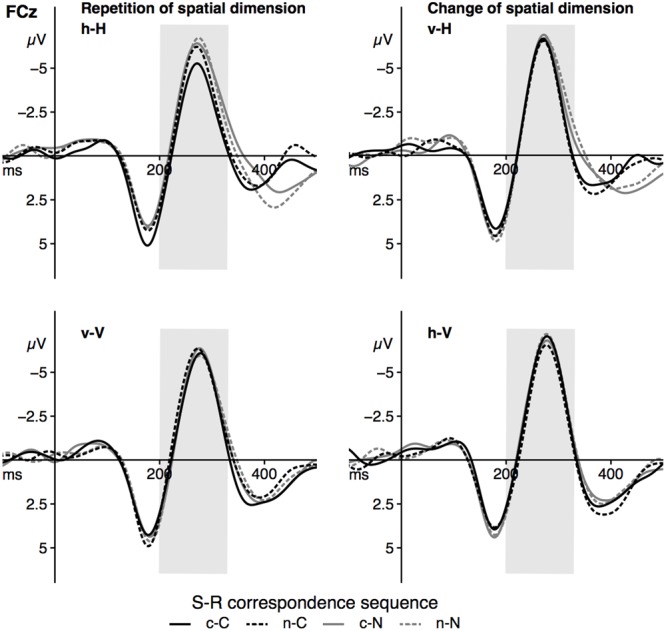
The figure displays the grand averages at electrode site FCz as a function of dimensional repetition (**left** panels) and dimensional change **(right)** as well as the current and previous S–R correspondence sequence (sequence types: c–C, n–C, c–N, n–N). Positive deflections are displayed downward. The gray area highlights the analyzed time-window of the N2 component (200–330 ms). Black lines signal current corresponding trials, gray lines signal current non-corresponding trials. Solid lines indicate previous corresponding and dashed lines indicate previous non-corresponding S–R relations. h = horizontal dimension, v = vertical dimension, c = corresponding, n = non-corresponding S–R relation. Small letters indicate previous trial features, capital letters current trial features of a trial sequence.

**FIGURE 5:**
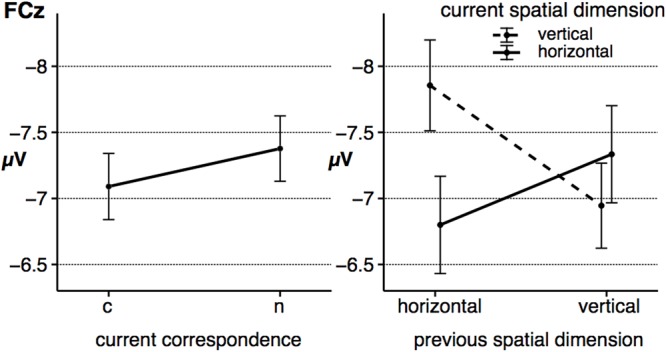
The figure shows the average peak amplitude of the fronto-central N2 component as a function of *corr N* **(left)** and as a function of *spat N* and *spat N-1* **(right)**. Error bars indicate the standard error of the mean. c = corresponding, n = non-corresponding S–R relations.

The grand averages at the electrode FCz is depicted in **Figure 4**, the fronto-central N2 peak amplitude as a function of the significant effects is displayed in **Figure 5**. For the peak amplitude of the fronto-central N2 component, a statistically significant main effect of the factor *corr N, F*(1,23) = 8.31, *p* = 0.008, $\eta_{p}^{2}$ = 0.27, signaled a spatial correspondence effect, that is a higher amplitude in non-corresponding trials compared to current corresponding trials (**Figure 5**, left panel). Furthermore, the interaction between *spat N* and *spat N-1, F*(1,23) = 28.64, *p* < 0.001, $\eta_{p}^{2}$ = 0.55, was also significant. Fronto-central N2 amplitudes were increased in sequences in which the spatial dimension changed compared to sequences without a change. This was evident for current horizontal trials, *F*(1,23) = 12.88, *p* = 0.002, $\eta_{p}^{2}$ = 0.36, as well as for current vertical trials, *F*(1,23) = 18.11, *p* < 0.001, $\eta_{p}^{2}$ = 0.44 (**Figure 5**, right panel).

With respect to the fronto-central N2 peak amplitude, no other main effect (*spat N*: *F*(1,23) = 2.27, *p* = 0.146, $\eta_{p}^{2}$ = 0.09, *corr N-1*: *F*(1,23) < 1, *spat N-1*: *F*(1,23) = 2.24, *p* = 0.148, $\eta_{p}^{2}$ = 0.09) and no other interaction (*corr N* × *spat N*: *F*(1,23) = 1.65, *p* = 0.212, $\eta_{p}^{2}$ = 0.07, *corr N* × *corr N-1: F*(1,23) < 1, *spat N* × *corr N-1*: *F*(1,23) = 1.21, *p* = 0.283, $\eta_{p}^{2}$ = 0.05, *corr N* × *spat N-1*: *F*(1,23) = 1.35, *p* = 0.257, $\eta_{p}^{2}$ = 0.06, *corr N-1* × *spat N-1*: *F*(1,23) < 1, *corr N* × *spat N* × *corr N-1*: *F*(1,23) = 1.05, *p* = 0.316, $\eta_{p}^{2}$ = 0.04, *corr N* × *spat N* × *spat N-1*: *F*(1,23) < 1, *corr N* × *corr N-1* × *spat N-1*: *F*(1,23) < 1, *spat N* × *corr N-1* × *spat N-1*: *F*(1,23) < 1, *corr N* × *spat N* × *corr N-1* × *spat N-1: F*(1,23) = 3.43, *p* = 0.077, $\eta_{p}^{2}$ = 0.13) reached significance.

### Parietal P3b Mean Amplitude

**FIGURE 6:**
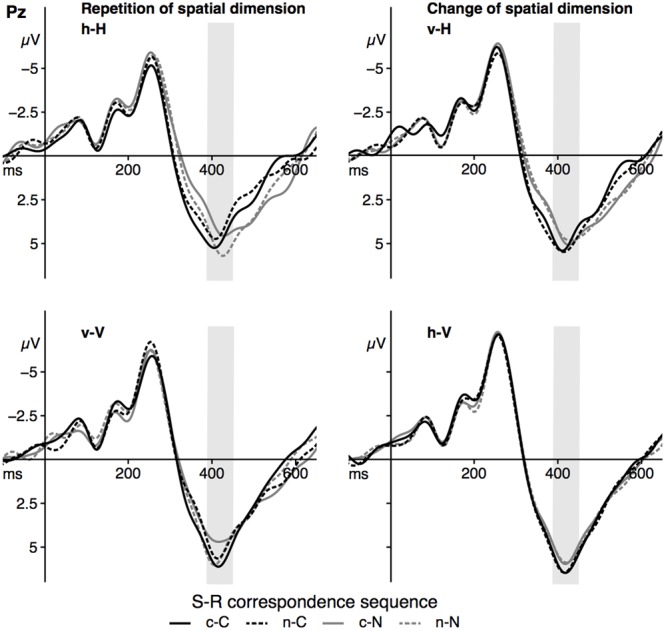
The figure displays the grand averages at electrode site Pz as a function of dimensional repetition **(left)** and dimensional change **(right)** and S–R correspondence sequence (sequence types: c–C, n–C, c–N, n–N). Positive deflections are displayed downward. The gray area highlights the analyzed time-window of the P3b component (390–450 ms). Black lines signal current corresponding trials, gray lines signal current non-corresponding trials. Solid lines indicate previous corresponding and dashed lines indicate previous non-corresponding S–R relations. h = horizontal dimension, v = vertical dimension, c = corresponding, n = non-corresponding S–R relations. Small letters indicate previous trial features, capital letters current trial features of a trial sequence.

**FIGURE 7:**
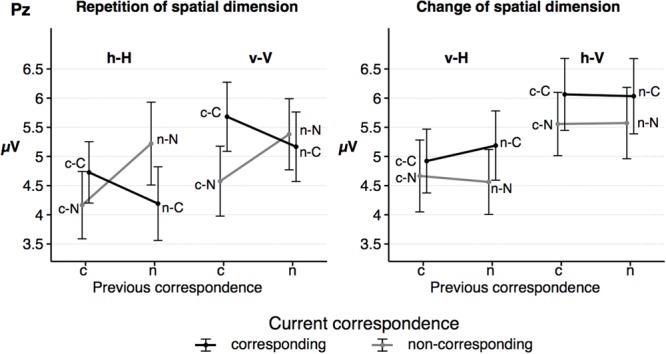
The figure shows the mean amplitude of the parietal P3b as a function of dimensional repetition **(left)** and dimensional change **(right)** and current and previous correspondence. Error bars indicate the standard error of the mean. h = horizontal dimension, v = vertical dimension, c = corresponding S–R relation, n = non-corresponding S–R relation. Small letters indicate previous trial features, capital letters current trial features of a trial sequence.

Grand averages for the P3b component at Pz are depicted in **Figure 6**, P3b mean amplitude for the sequence conditions in **Figure 7**. The ANOVA showed a significant main effect of the factor *spat N, F*(1,23) = 35.26, *p* < 0.001, $\eta_{p}^{2}$ = 0.61. P3b mean amplitude was more pronounced in vertical trials compared to horizontal trials. As indicated by the significant interaction of *spat N* and *spat N-1, F*(1,23) = 19.80, *p* < 0.001, $\eta_{p}^{2}$ = 0.46, a change of the spatial dimension only had an effect on the P3b on current vertical trials, *F*(1,23) = 20.06, *p* < 0.001, $\eta_{p}^{2}$ = 0.47, but not on current horizontal trials, *F*(1,23) = 2.93, *p* = 0.100, $\eta_{p}^{2}$ = 0.11. In the vertical dimension, the P3b amplitude was increased after a change of the spatial dimension compared to a repetition of the dimension.

Furthermore, the interaction of *corr N* and *corr N-1* indicated a sequential modulation of the P3b mean amplitude, *F*(1,23) = 9.42, *p* = 0.005, $\eta_{p}^{2}$ = 0.29. Only after spatially corresponding trials, *F*(1,23) = 11.65, *p* = 0.002, $\eta_{p}^{2}$ = 0.34, but not after non-corresponding trials, *F*(1,23) < 1, a spatial correspondence effect on the P3b was found, with increased P3b amplitudes in currently corresponding compared to currently non-corresponding trials. Importantly, the fourfold interaction of *corr N, spat N, corr N-1* and *spat N-1, F*(1, 23) = 11.39, *p* = 0.003, $\eta_{p}^{2}$ = 0.33, indicated that this sequential modulation of the P3b amplitude occurred only in h–H sequences, *F*(1,23) = 12.91, *p* = 0.002, $\eta_{p}^{2}$ = 0.36, but not in v–H sequences, *F*(1,23) = 2.88, *p* = 0.103, $\eta_{p}^{2}$ = 0.11, and in current vertical sequences, *F*(1,23) = 3.98, *p* = 0.058, $\eta_{p}^{2}$ = 0.15. For h–H sequences, the result pattern was as follows: after spatially corresponding trials the P3b was increased on currently corresponding trials compared to on currently non-corresponding trials, *F*(1, 23) = 4.41, *p* = .047, $\eta_{p}^{2}$ = 0.17, whereas after non-corresponding trials the P3b was increased on currently non-corresponding trials compared to currently corresponding trials, *F*(1,23) = 5.97, *p* = 0.023, $\eta_{p}^{2}$ = 0.21. In other words, P3b amplitudes were increased in trial sequences, in which the correspondence condition was repeated compared to trials where the correspondence condition changed (see **Figure 7**). For the P3b amplitude, no other main effect (*corr N*: *F*(1,23) = 3.79, *p* = 0.064, $\eta_{p}^{2}$ = 0.14, *corr N-1*: *F*(1,23) = 1.03, *p* = .32, $\eta_{p}^{2}$ = .04, *spat N-1*: *F*(1,23) = 2.73, *p* = 0.112, $\eta_{p}^{2}$ = 0.11) and no other interaction (*corr N* × *spat N*: *F*(1,23) = 1.63, *p* = 0.215, $\eta_{p}^{2}$ = 0.07, *spat N* × *corr N-1*: *F*(1,23) < 1, *corr N* × *spat N-1*: *F*(1,23) = 2.48, *p* = 0.129, $\eta_{p}^{2}$ = 0.1, *corr N-1* × *spat N-1*: *F*(1,23) < 1, *corr N* × *spat N* × *corr N-1*: *F*(1,23) < 1, *corr N* × *spat N* × *spat N-1*: *F*(1,23) = 3.29, *p* = 0.083, $\eta_{p}^{2}$ = 0.13, *corr N* × *corr N-1* × *spat N-1*: *F*(1,23) = 1.45, *p* = 0.241, $\eta_{p}^{2}$ = 0.06, *spat N* × *corr N-1* × *spat N-1*: *F*(1,23) < 1) was significant.

## Discussion

The present study aimed to investigate the contribution of two supposed mechanisms of sequential modulations of the Simon effect, that is conflict adaptation effects (Botvinick et al., 2001; Stürmer et al., 2002) and feature integration effects (Hommel, 1998; Hommel et al., 2004). To this end, we used a spatially two-dimensional Simon task, that is the stimuli could appear vertically or horizontally, with vertically arranged response buttons. This study design allowed us to analyze sequential modulations of the Simon effect in the horizontal as well as in the vertical dimension, but without task-switching or a change of the relevant stimulus dimension (cf. Egner, 2008; Notebaert and Verguts, 2008). In order to gain a deeper understanding of the processes involved, we recorded EEG and analyzed ERP components reflecting relevant processes connected to conflict adaptation (N2) and feature integration effects (P3b).

According to the conflict adaption account (Botvinick et al., 2001; Stürmer et al., 2002), the size of the Simon effect should be a function of the previous and current correspondence conditions irrespective of changes of the spatial dimension. In contrast, the feature integration account (Hommel, 1998; Hommel et al., 2004) posits that a repetition or alternation of the spatial dimension should be critical. An alternation of the spatial dimension produces a complete change of task features for half of these trials and a partial repetition for the other half, which makes an unbinding process necessary. Importantly, as this pattern is the same for any S-R correspondence sequence (c–C, c–N, n–C, n–N), the feature integration account predicts no sequential modulation of the Simon effect when the spatial dimension changes.

We found an overall Simon effect and an overall spatial S-R correspondence effect on accuracy, however, the size of the Simon effect was larger in the horizontal dimension compared to the vertical dimension (cf. Nicoletti and Umiltà, 1984). Thus, although the instruction only emphasized the vertical S–R mapping (e.g., press the upper key in response to the letter X and the lower key in response to the letter S), the implied horizontal S–R mapping between response hand and stimulus dimension modulated performance as well and created a Simon effect.

The observed RT data is perfectly in line with the predictions of the feature integration account (Hommel, 1998; Hommel et al., 2004), as any sequential modulations of the Simon effect, which were evident within both spatial dimensions, were eliminated after a change of the spatial dimension (see also Lee and Cho, 2013). Within each spatial dimension, that is in h–H and in v–V trial sequences, the Simon effect was eliminated (horizontal dimension) or reversed (vertical dimension) after non-corresponding relative to after corresponding trials. This pattern of results is also evident for the accuracy data. The RT analyses conducted for the neutral trials further corroborate this pattern which suggests that it might be not a preceding conflict situation per se, which sequentially modulates the size of the Simon effect and questions the assumptions of the conflict adaptation account (Botvinick et al., 2001; Stürmer et al., 2002). However, other studies found evidence for the conflict adaptation effects while controlling for feature integration effects (e.g., Wühr, 2005). Wühr (2005) introduced a second spatial dimension in order to create specific types of stimuli that allowed to force unbinding processes when the correspondence condition repeated. In contrast to our study, however, stimuli always featured horizontal as well as vertical information. This was also the case in the study of Braem et al. (2011), who found sequential effects across spatial dimensions.

Similar to the RT data, the size of the parietal P3b amplitude was only sequentially modulated when the spatial dimension was repeated. In this case, the amplitude of the P3b component was larger in sequences in which the correspondence condition was repeated (c–C, n–N) compared to sequences in which it alternated (c–N, n–C). Only the latter includes partial repetitions, which require an unbinding of the previous event file before creating a new one. Thus, situations with a supposedly easier response selection, that is sequences without unbinding processes, elicited larger P3b amplitudes. Importantly, sequences with a change of the spatial dimension, in which unbinding processes are equally likely for all correspondence sequences, did not show such sequential modulations on the P3b. Assuming that the parietal P3b might reflect requirements on response selection processes and some sort of reactivation processes concerning S–R links (Verleger et al., 2014), this pattern seems to corroborate the influence of feature integration effects. However, although the descriptive data pattern was very similar in both spatial dimensions, the P3b effect was only statistically significant in h–H sequences but not in v–V sequences. The non-significant effect for the vertical dimension might be due to differences in the two spatial dimensions, which will be discussed later in this section. In contrast to the P3b amplitude, the fronto-central N2 component showed a Simon-like effect. In the present study, the N2 amplitude was increased on current non-corresponding compared to current corresponding trials. This effect is mirrored in the increased RT and error rates on non-corresponding trials relative to corresponding trials (see also Chen and Melara, 2009) reflecting increasing cognitive control demands (e.g., Folstein and Van Petten, 2008). Alternatively, it might reflect the increased RTs themselves (cf. Grinband et al., 2011).

The conflict adaptation account predicts lower conflict after non-corresponding trials. The preceding conflict in the Simon task should boost cognitive control and thus decreases the influence of irrelevant information. After corresponding trials, the direct path stays open on corresponding trials as the irrelevant spatial information it provides reinforces the response tendencies activated by the indirect path and is thus beneficial on these types of trials. Hence, the flow of irrelevant information is not reduced in the following trial (Botvinick et al., 2001; Stürmer et al., 2002). In accordance with these assumptions, some previous research has found the fronto-central N2 amplitude to be modulated sequentially in an SRC task (Clayson and Larson, 2011). However, in line with other earlier studies (e.g., Wendt et al., 2007) we did not find any conflict adaptation effects on the fronto-central N2 amplitude. Previous research has already indicated that the transfer of control may depend on the conflict-type (Egner et al., 2007) and the similarity of the employed tasks (e.g., Braem et al., 2014). Such argument does not apply to the present study as conflict-type, the relevant stimulus dimension (see Notebaert and Verguts, 2008) and the S-R mapping were the same for both spatial dimensions and we did not include any task-switching elements (cf. Egner, 2008). The current experimental setup was thus designed to promote the emergence of conflict adaptation. Yet, the current fronto-central N2 pattern matches the behavioral data, which shows feature integration rather than conflict adaptation effects. On the other hand, the fronto-central N2 may be relatively insensitive to such transfer effects (cf. Wendt et al., 2007).

Alternatively, conflict adaptation effects on the fronto-central N2 may have been obscured by the introduction of a second spatial dimension: Instead of a sequential modulation of the fronto-central N2 amplitude we observed a “spatial sequence effect” as the amplitude of the N2 peak was enhanced when the spatial dimension changed from one trial to the next. It is feasible that a change of the spatial dimension would increase the need for a more controlled processing mode. In this respect, the switch between the two spatial dimensions might bear similarities to the switch between different tasks. Akin to a task switch, a switch between spatial dimensions may hinder the transfer of control and thus obscure the effects of conflict adaptation on fronto-central N2 amplitude. As mentioned earlier, different task-specific factors are supposed to influence the trial-to-trial transfer of control (for reviews, see e.g., Egner, 2008; Braem et al., 2014). For example, studies combining different SRC tasks could not observe a transfer of control across the SRC tasks when the conflict-type differed (e.g., Egner et al., 2007). Furthermore, Notebaert and Verguts (2008) found a mutual influence of control between different SRC tasks, but only when the relevant stimulus dimension was kept identical, which was also the case in the present study. The present result pattern might point toward a potential limitation of our study. There may be fundamental differences to the manner in which the two spatial dimensions are treated in the human information processing system and these differences may mask relevant modulations of the Simon effect. In keeping with this, it is still under debate whether the vertical and the horizontal Simon effect may have different underlying mechanisms (Vallesi et al., 2005; Wiegand and Wascher, 2005). In the present study, quantitative differences in behavioral Simon effects as well as the P3b data pattern, that is the fact that the sequential modulation of the P3b was only statistically significant for the horizontal dimension, corroborate the notion that the vertical and the horizontal Simon effect may be different (but see Nicoletti and Umiltà, 1984).

With respect to the observed differences in information processing between the vertical and the horizontal dimension, one might also speculate that both spatial dimensions elicited dimension-specific control mechanisms, which do not modulate conflict processing in the other spatial dimension (for a review about the specificity of conflict adaptation see, e.g., Braem et al., 2014). However, our analysis of fronto-central N2 amplitude did not yield a fourfold interaction, that is sequential modulation effects within each spatial dimension, which might be interpretable as evidence for different dimension-dependent control mechanisms. Note that such a fourfold interaction could also be seen as evidence in support of feature integration effects: As previous research indicates, fronto-central N2 amplitude may be sensitive to changes of task features and thus might be modulated by unbinding processes (Chen and Melara, 2009). Assuming an increased need for controlled processing in sequences, which require an unbinding process, these sequences should evoke larger fronto-central N2 amplitudes compared to sequences in which no such unbinding process is necessary. The present N2 result pattern does not conform to these assumptions of the feature integration account, however, as there is also no sequential modulation when the spatial dimension is repeated and the proportion of partial repetitions is unequal between the different correspondence sequences.

To evaluate the predictions of the feature integration account on a more fine-grained level, we reanalyzed our behavioral data in terms of complete repetitions, complete alternations and partial repetitions separately for both spatial dimensions (cf. Hommel et al., 2004). In light of the sequential analyses we performed, the amount of trials per condition was too small, however, to conduct a similar analysis of the EEG data with appropriate power. For RT as well as for accuracy, we found an interaction of stimulus location repetition and stimulus identity (and response) repetition in both spatial dimensions, which indicate better performance, when either all task features repeat or change. Yet, for RT data we further found main effects of both factors, which contradict the feature integration assumptions, because responses to full changes were much slower than responses in full repetition trials. The feature integration account, however, would assume that responses in full change trials are as efficient as in full repetition trials. Thus, as our overall result pattern indicates: There is evidence in favor of the feature integration account, but it is not unambiguous. To control for factors, which might impede the transfer of control, among other things we used the same S–R mapping for the vertical and horizontal dimension. This course of action might also have its drawbacks, however, as the joint representation of S–R associations might introduce a binding across spatial dimensions, which would otherwise not exist (i.e., with different responses for stimuli on the two spatial dimensions).

Overall, we found evidence for an increased need for control in current non-corresponding S–R situations, but the fronto-central N2 amplitude in our design did not indicate any conflict adaptation effects or transfer of control. However, the fronto-central N2 might be insensitive with respect to conflict adaptation effects (cf. Wendt et al., 2007, but see Clayson and Larson, 2011). Nevertheless, our behavioral data indicated a reduced deterioration in performance on non-corresponding trials, when the preceding trial also involved a response conflict. As this effect was only evident when subsequent trials were on the same spatial dimension, the result pattern is in line with the idea that feature integration effects at least contribute to sequential modulations of spatial correspondence effects in the Simon task. P3b data are also more in line with the feature integration account as there was no sequential modulation of the P3b after an alternation of the spatial dimension. We found evidence that the P3b amplitude might be sensitive to feature integration effects. Our result pattern, however, may also be due to repetition priming effects (see Mayr et al., 2003) that cannot be analyzed reliably in the present data due to a too low number of available trials. Recent accounts on sequential effects in SRC tasks additionally propose multi-level learning effects, including abstract learning with respect to control mechanisms and concrete learning with respect to task features (e.g., Verguts and Notebaert, 2008; Weissman et al., 2015). As for example, physiological states like arousal may modulate the sequential integration of task features (Verguts and Notebaert, 2008). Also, RT carry-over effects may contribute to sequential effects in SRC tasks (cf. Huber-Huber and Ansorge, 2016). However, mathematical modeling have shown that at least the latter can only explain parts of the effects observed (Huber-Huber and Ansorge, 2016), that might be driven by one of the before mentioned core mechanisms. Thus, our result pattern provides evidence that for sequential modulations in the Simon task memory effects like unbinding processes might be more relevant than conflict adaptation effects.

## Author Contributions

EW and KH devised the experimental design and analyzed the data. KH collected the data and wrote the manuscript. The interpretation, revision, and final editing of the work were conducted by EW, KK, and KH.

## Conflict of Interest Statement

The authors declare that the research was conducted in the absence of any commercial or financial relationships that could be construed as a potential conflict of interest.
